# High Interfacial Adhesion of PET/Rubber Composites by a New Eco-Friendly Dipping System

**DOI:** 10.3390/polym18030338

**Published:** 2026-01-27

**Authors:** Aolian Wu, Yanlin Liu, Tong Sun, Mei Shen

**Affiliations:** 1 Key Laboratory of Rubber-Plastics, Ministry of Education, School of Polymer Science and Engineering, Qingdao University of Science and Technology, Qingdao 266042, China; wal7693@163.com (A.W.); st05972@163.com (T.S.); 2 Shandong Provincial Key Laboratory of Rubber-Plastics, School of Polymer Science and Engineering, Qingdao University of Science and Technology, Qingdao 266042, China; 3 College of Engineering, Peking University, Beijing 100871, China; 2300011185@stu.pku.edu.cn

**Keywords:** polyester fiber, KH550, rubber, environmentally friendly impregnation

## Abstract

Fiber-reinforced rubber composites (FRRC) are widely employed in critical industries, such as the automotive, aerospace, and construction protection industries, due to their excellent deformation resistance and superior mechanical properties. Polyester (PET) fiber, with its outstanding dimensional stability and cost-effectiveness, has increasingly replaced nylon as the primary reinforcement in radial tires. However, the lack of polar groups on PET surfaces results in poor interfacial adhesion with rubber matrices, limiting composite performance. Traditional resorcinol–formaldehyde–latex (RFL) dipping systems enhance adhesion but raise environmental and health concerns due to the release of hazardous substances. This study develops a novel eco-friendly γ-Aminopropyltriethoxysilane (KH550)–glycerol triglycidyl ether–sorbitol glycidyl ether–2-Ethyl-4-methylimidazole–latex (KG-SML) dipping system to enhance PET–rubber interfacial adhesion. At an optimal KH550 dosage of 2 phr, the 180° peel force and H pull-out force reached maximum values of 23.5 N/piece and 109.0 N, respectively, significantly surpassing the performance of the conventional RFL system. The KG-SML system offers an effective and sustainable alternative to RFL, with enhanced interfacial performance and less environmental impact.

## 1. Introduction

Fiber-reinforced rubber composites (FRRC) combine the high strength and modulus of fiber skeleton materials with the high resilience of the rubber matrix, endowing the composite with exceptional resistance to deformation and significant advantages in terms of mechanical properties and processability [1,2]. Consequently, FRRCs play a crucial role in various industries such as the automotive, aerospace, and construction protection industries [3]. Polyester fiber (PET) has gradually replaced traditional nylon cord as the reinforcing framework material for radial tires, primarily due to its excellent dimensional stability and significantly lower production costs compared to those of nylon. However, the molecular structure of polyester fiber presents a critical limitation: it contains no polar groups aside from two terminal hydroxyl groups [4]. This structural feature results in low surface activity, which in turn leads to poor interfacial adhesion between the fiber and the rubber matrix. In practical applications, such inadequate interfacial bonding can cause issues like delamination and slippage in rubber products, ultimately compromising the product’s performance and durability [5,6,7].

Therefore, in industrial production, the modification of fiber is crucial [8,9,10]. Common modification strategies for modification include increasing the surface roughness of fiber or introducing reactive groups onto its surfaces, both aimed at enhancing interfacial adhesion with rubber [11,12,13,14,15]. Current methods employed for fiber surface modification include plasma treatment [16,17,18,19], ultrasonic treatment [20], γ-ray irradiation, ultraviolet irradiation [21,22,23], and electron beam irradiation. However, these methods are associated with relatively high costs and complex processing procedures. Furthermore, they tend to damage the surface structure of the fibers, which impairs the fibers’ service performance. Currently, the industry commonly employs the technologically mature and low-cost resorcinol–formaldehyde–rubber latex (RFL) dipping system for fiber surface modification [1]. The phenolic resin structure in this system forms covalent bonds with functional groups on the fiber surface, enhancing the adhesion of the fiber surface. Simultaneously, the latex particles dispersed within the resin network undergo co-crosslinking reactions with the rubber matrix during vulcanization, further strengthening the interfacial adhesion between the fiber and rubber, thereby improving the mechanical properties and durability of the composite [24,25]. However, the RFL system also presents potential health and environmental risks. Both resorcinol and formaldehyde in the RFL system are harmful to human health and the environment. The World Environmental Protection Agency (EPA) and the International Agency for Research on Cancer (IARC) have classified formaldehyde and resorcinol as potential human carcinogens.

Thus, the developing of more environmentally friendly non-RFL dipping systems is of significant importance for building an environmentally friendly society [26,27,28]. Li et al. [29] designed a novel eco-friendly carbon-reducing dipping system (PI-EP-LGTL) for the modification of polyimide fibers. The polyimide fibers were first activated with blocked diisocyanate and epoxy resin, followed by secondary coating with an impregnating solution composed of vinylpyridine (VP) latex, lignin, tannic acid (TA) and glyoxal. Zhang et al. [30] designed an eco-friendly dipping and bonding system composed of epoxy resin, amine curing agent and latex for polyester fibers, and adopted the direct coating method to modify the surface of polyester fibers. After the epoxy resin was cured by amino groups, a robust resin layer was formed between the fibers and rubber. Experimental results show that the bonding performance of the composite can reach 90% of that of the traditional RFL dipping system.

In this study, based on a novel environmentally friendly dipping system (G-SML system), a reinforcement system was selected. First, PET fibers were activated using an ethanol solution of glycerol triglycidyl ether (GTE) to introduce hydroxyl and epoxy groups onto the fiber surface. Subsequently, secondary dipping was performed using an impregnation solution containing 2-ethyl-4-methylimidazole (MZ), sorbitol glycidyl ether (SGE), and styrene–butadiene–vinylpyridine (VP) latex. The resin structure forms an interpenetrating network with the molecular chains of the VP latex dispersed within it. The VP latex physically entangles and chemically co-vulcanizes with the rubber matrix during vulcanization, achieving interfacial adhesion between the polyester cord and rubber. To enhance the adhesive bonding capability, two methods were investigated: (1) activation of the polyester fibers by incorporating a silane coupling agent into the first dipping solution, and (2) reinforcement by adding fillers into the second dipping solution. However, due to the challenge of poor filler dispersion, surface activation of the PET substrate was ultimately performed using silane coupling agents to enhance interfacial adhesion. In the first dipping system, γ-Aminopropyltriethoxysilane (KH550) was added. KH550 reacts with PET fibers, introducing amino groups (-NH_2_) as reactive groups onto the fiber surface. This modification enhances the reactivity of PET fibers and improves the interfacial adhesion performance between PET fibers and the rubber matrix.

## 2. Materials and Methods

### 2.1. Materials

Polyester (PET) fibers were sourced from Qingdao Tianbang New Materials Co., Ltd., Qingdao, China, with a specification of 1100 dtex/2 × 3 and 1100 dtex/1 × 3. Styrene–butadiene–vinylpyridine (VP) latex was obtained from Jiangsu Yatai Chemical Co., Ltd., Nantong, China. Glycerol triglycidyl ether (GTE) was acquired from Beijing Hanlongda Science and Technology Development Co., Beijing, China. Sorbitol glycidyl ether (SGE) was purchased from Wuhan Lanabai Pharmaceutical Chemical Co., Ltd., Wuhan, China. 2-Ethyl-4-methylimidazole (MZ) was purchased from Shanghai Macklin Biochemical Technology Co., Ltd., Shanghai, China. Sodium montmorillonite (Na-MMT) was procured from Shanghai Macklin Biochemical Technology Co., Ltd. Carbon black N220, Styrene-butadiene rubber (SBR), γ-Aminopropyltriethoxysilane (KH550), ZnO, stearic acid, and bis-(γ-triethoxysilylpropyl)-tetrasulfide (Si-69) were all commercially available products. The composition of the SBR compound employed in this study is detailed in Table 1.

### 2.2. Preparation of Dipping Solution

The first dipping solution was formulated using GTE and silane coupling agents.

Under controlled temperature conditions of 25 °C, GTE and silane coupling agents were combined in a mass ratio of 1:9 and stirred continuously for 30 min until the GTE was completely dissolved in the anhydrous ethanol, forming a uniform-base first dipping solution. Subsequently, silane coupling agents Si-69 and KH550 were added separately to the prepared base dipping solution, followed by continued stirring for an additional 10 min to ensure that the silane coupling agents were fully dissolved and uniformly dispersed within the system. This process ultimately yielded two distinct one-bath dipping solution formulations.

The second dipping solution was prepared using SGE–MZ–VP latex. Under a controlled temperature of 25 °C, SGE and MZ (with a ratio of SGE/MZ = 2/5) were mixed with 50 g of deionized water and stirred continuously for 0.5 h. Subsequently, VP latex was added, and stirring continued for an additional hour until no oil droplets were observed at the bottom of the liquid. This process resulted in the preparation of the SML green two-bath base dipping solution. Subsequently, an aqueous solution of Na–MMT or an aqueous dispersion of GO was individually introduced into the prepared base dipping solution. This was followed by continuous stirring for 20 min to ensure that the fillers were completely dissolved and uniformly dispersed within the system, ultimately resulting in two distinct formulations of two-bath dipping solutions.

### 2.3. Dip-Coating of PET Fiber Cords

The PET fibers were initially dried in an oven at 60 °C for 120 min prior to use. Subsequently, the PET fiber cords underwent dip-coating utilizing a dipping machine. The conditions for dipping were as follows: first, the fibers were dried at 150 °C for 100 s; second, they underwent an additional drying phase at 150 °C for another 100 s; and finally, they were cured at 230 °C for a period of 100 s.

### 2.4. Preparation of PET Fiber/Rubber Composites

**Figure 1:**
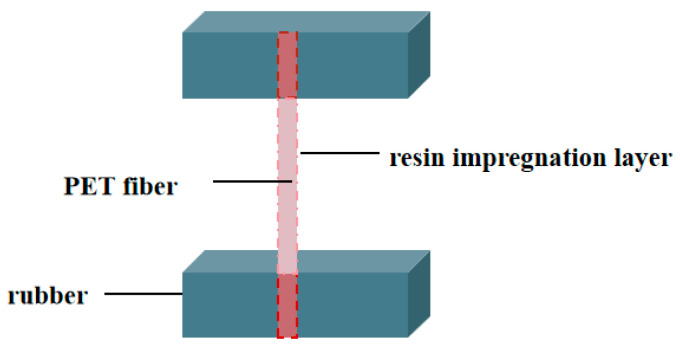
Diagram of H pull-out sample.

The preparation of H pull-out test samples was conducted in accordance with the standard [31]. The compounded rubber was cut into strips measuring 200 mm × 10 mm × 4 mm. Four strips were positioned within the rubber slots of both the upper and lower plates of the mold. The treated polyester fibers were secured in the mold slots under tension. Subsequently, the mold was closed and placed in a plate vulcanizing press. The vulcanization temperature was set at 160 °C, with a vulcanization time of t90 + 4 min. A pressure of 15 MPa was applied during the vulcanization process. A schematic diagram illustrating the H pull-out test specimen for the fiber/rubber composite is presented in Figure 1.

**Figure 2:**
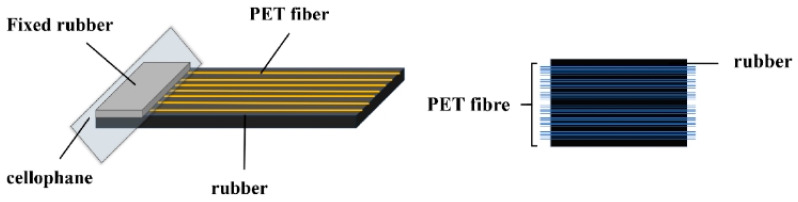
Diagram of stripped sample.

The preparation of the 180° peel test samples was conducted in accordance with the standard [32]. The treated PET fibers were fixed within the mold slots under tension. Subsequently, layers of fixed rubber sheets, isolating cellophane, and test rubber sheets were arranged sequentially. The mold was then closed and placed into a plate vulcanizing press. The vulcanization temperature was set at 160 °C, with a vulcanization time of t90 + 2 min. A pressure of 15 MPa was applied during the vulcanization process. A schematic diagram illustrating the configuration of the 180° peel test specimen for the fiber/rubber composite is presented in Figure 2.

### 2.5. Characterization

Differential Scanning Calorimetry (DSC-3, METTLER TOLEDO, Greifensee, Switzerland) was used to test the reaction heat effects of the samples. The testing conditions were as follows: a nitrogen atmosphere, temperature range of 30–200 °C and heating rate of 25 °C/min. Attenuated Total Reflectance Fourier Transform Infrared Spectroscopy (ATR-FTIR) (VERTEX70 Fourier Transform Infrared Spectroscopy, North Billerica, MA, USA) was employed to characterize the chemical structural changes in the fibers and the dip solution before and after dipping. The scanning range was from 400 cm^−1^ to 4000 cm^−1^, with a spectral resolution of 4 cm^−1^ and 32 scans. X-Ray Photoelectron Spectroscopy (XPS) was performed on a photoelectron spectrometer (Thermo Fisher Science and Technology, Waltham, MA, USA) to analyze the elemental composition of the polyester fiber surface before and after dipping. The microscopic morphology of PET fibers after peeling from the rubber was characterized using Scanning Electron Microscopy (SEM, JEOL JSM-6700F, Tokyo, Japan). Samples were sputter-coated with gold and tested at an accelerating voltage of 30 kV. The H pull-out of the adhesion between the dipped cords and rubber was tested according to the standard [31] using a tensile tester (GT-TCS−200, Gotech Testing Machines Inc., Taichung, Taiwan, China). The test speed was set to (100 ± 10) mm/min. Each test used five samples. The 180° peel of the adhesion between the dipped cords and rubber was tested according to the standard [32].The test speed was set to (300 ± 10) mm/min. Each test used five samples. The tensile strength of the fibers before and after dipping was tested according to the standard [33]. The tensile speed was (100 ± 10) mm/min. Each test used five samples.

## 3. Results and Discussion

### 3.1. Dip-Coating Principle

To investigate the impact of fiber pretreatment and dipping processes on the interfacial adhesion properties of fiber/rubber composites, this study designed a series of comparative experiments. Scheme 1 involved incorporating two different silane coupling agents, KH550 and Si-69, into the formulation of the first dipping solution within the G-SML system, while ensuring consistency in the traditional G-SML second dipping solution. Scheme 2 involved utilized a GTE ethanol solution as the first dipping solution and introduced Na-MMT and GO into the second dipping solution for reinforcement.

### 3.2. Effect of Dipping Systems on the Interfacial Adhesion of Fiber/Rubber Composites

**Figure 3:**
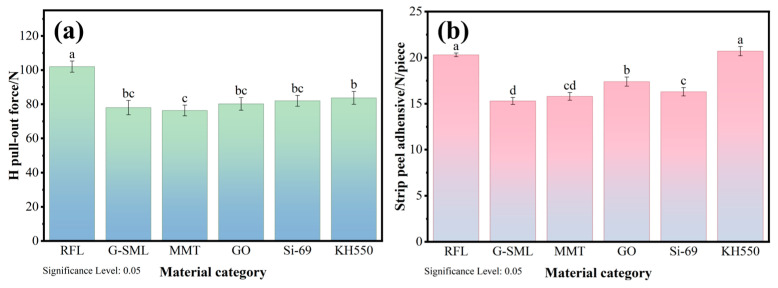
(**a**) H pull-out test and (**b**) 180° peel test of PET/SBR composites modified by different materials (Same lowercase letters indicate no significant difference between two groups of data, while different lowercase letters indicate a significant difference between them).

To specifically illustrate the impact on the interfacial bonding between the fiber and rubber, 180° peel and H pull-out tests were conducted. The relationship among the 180° peel force, H pull-out force, and the type of materials is presented in Figure 3. The results indicate that the enhanced adhesive performance of KH550 was significantly superior to that of other treatments. This discrepancy arises from the fundamental differences in their mechanisms of action: silane coupling agents primarily fortify the interface through chemical bonding, whereas nanofillers predominantly rely on physical adsorption. The amino group present in KH550 functions as a nucleophile, attacking the electron-deficient ester group within PET fibers, which results in the ammonolysis of the PET. This process facilitates the grafting of covalent amide bonds onto the surface of PET fibers, effectively transforming their initially inert surface into an active amino-functionalized one. Concurrently, hydroxyl groups are generated on the fiber surface through a reaction with glycerol ether in the first bath, allowing silane coupling agents to react with these hydroxyl groups and form covalent bonds. The chemical interaction between Si-69 and PET is notably weak. Unlike the amino groups in KH550, Si-69 cannot form strong chemical bonds or hydrogen bonds with the terminal groups or ester groups of PET. Its adhesion to the fibers primarily relies on physical adsorption and weak siloxane bonding. Due to their extremely high specific surface area and surface energy, nanofillers are prone to agglomeration, making it difficult to achieve stable and uniform dispersion in the dipping solution. Moreover, the lack of active functional groups on the surface of nanofillers results in poor compatibility with polyester fibers, which hinders the formation of strong interfacial bonding. Consequently, nanofillers offer limited enhancement to the adhesive performance of the dipping system.

### 3.3. Chemical Structure of the Dip-Coated Fibers Surface

**Figure 4:**
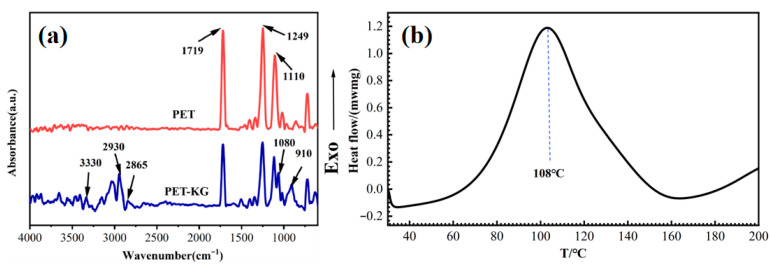
(**a**) ATR-FTIR spectra of fiber surface; (**b**) DSC curves of MZ/SGE = 5/2 curing reaction.

Since the modification effect of KH550 is the best, we will conduct further research on this scheme. Chemical structural changes on the fiber surface before and after dipping were characterized by ATR-FTIR measurements. Figure 4a shows the ATR-FTIR spectra of pristine PET fiber and PET-KG fiber (treated with GTE and KH550). The spectrum of untreated PET fiber shows characteristic absorption peaks at 1719 cm^−1^ (attributed to C=O stretching vibration), and at 1249 cm^−1^ and 1110 cm^−1^ (attributed to C-O-C stretching vibration). After dip-coating treatment, new broad bands appeared in the spectrum of PET-KG fiber around 3320 cm^−1^ (hydroxyl group), 2930 cm^−1^, 2865 cm^−1^ (methylene stretching vibrations), and 910 cm^−1^ (epoxy group), indicating successful impregnation of GTE onto the PET fiber. Additionally, the appearance of a characteristic C-O-Si absorption peak at 1080 cm^−1^ indicates the successful introduction of KH550. The DSC results shown in Figure 4b indicate that the curing reactions of SGE and MZ begin at approximately 60 °C, reaching an exothermic peak at around 108 °C. This temperature marks the point where the cross-linking reaction occurs most rapidly. Based on these findings, appropriate drying and curing temperatures can be selected.

**Figure 5:**
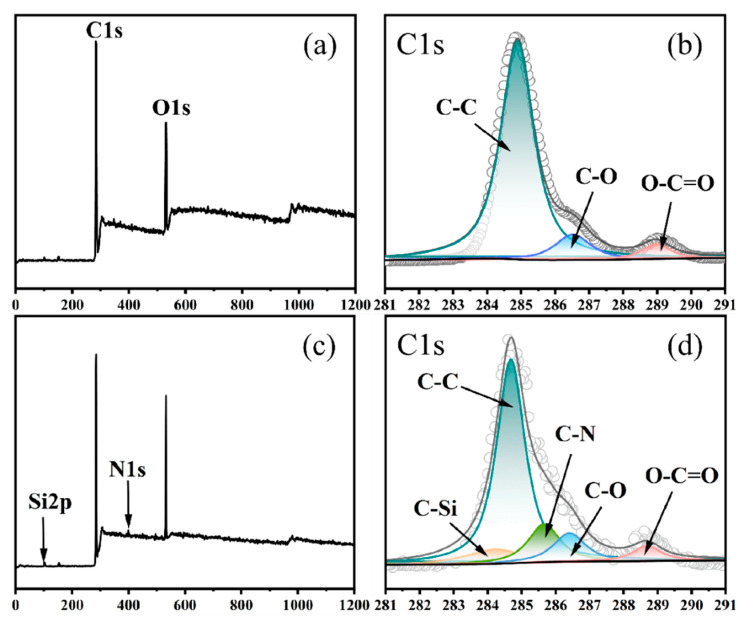
XPS spectra of PET fibers (**a**,**b**); PET-KG fiber (**c**,**d**) after KH550 one-bath treatment; (**a**,**c**) wide scan spectrum; (**b**,**d**) C1s nuclear energy level spectrum.

X-Ray Photoelectron Spectroscopy (XPS) was used to further observe the influence of dip-coating treatment on the chemical structure of the fiber surface. Figure 5 shows the XPS wide scan spectra and C1s core-level spectra of unmodified PET fiber and PET-KG fiber after the first bath activation. As shown in Figure 5a, the XPS wide scan spectrum of PET fiber shows two distinct peaks corresponding to C1s at 283.1 eV and O1s at 534.1 eV. After the first bath treatment, new peak components appear in the spectrum of PET-KG fiber (Figure 5c) at binding energies (BE) of approximately 100 eV and 400 eV, which can be attributed to the introduction of the silicon element from KH550. The atomic concentrations of carbon, oxygen, and nitrogen on the surface of the PET fiber before dipping were 83.7%, 15.1%, and 1.2%, respectively. After the first bath dipping, the oxygen atomic content on the PET-KG fiber surface increased due to the abundance of epoxy groups in the GTE structure. Simultaneously, due to the introduction of KH550, the surface contents of nitrogen and silicon atoms increased. As shown in Figure 5b, the C1s core-level spectrum of the PET fiber can be deconvoluted into three peak components: the C-C peak (~284.5 eV), C-O peak (~286.1 eV), and O-C=O peak (~288.6 eV). These bonds originate from the carbon chain and ester group structures of the PET fiber. As shown in Figure 5d, compared to that of the PET fiber, the area of the C-O peak increased in the PET-KG fiber, indicating successful grafting of GTE onto the PET fiber surface. This is consistent with the increase in the C-O-C absorption peak at 1110 cm^−1^ in the ATR-FTIR results (Figure 4a in original). Meanwhile, new C-N and C-Si peaks appeared on the surface of the PET-KG fiber, both attributable to the introduction of KH550.

In summary, these changes observed in the XPS and ATR-FTIR results indicate that the dipping solution successfully introduced active functional groups onto the fiber surface through curing reactions. The dense impregnation layer formed by the reaction created a bridge for adhesion between the fiber and rubber.

### 3.4. Influence of KH550 Content on the Static Adhesion Properties of Fiber/Rubber Composites

**Figure 6:**
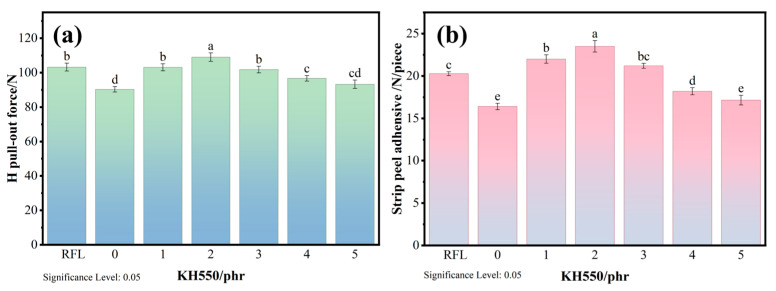
(**a**) H pull-out test and (**b**) 180° peel test of PET/ SBR composites with different KH550 contents (Same lowercase letters indicate no significant difference between two groups of data, while different lowercase letters indicate a significant difference between them).

Figure 6 reveals the influence of KH550 content on the adhesion properties of the KG-SML dipping system-coated PET/SBR composites. As shown in the figure, with increasing KH550 content, the H pull-out force and peel force of the KG-SML system-modified fiber/rubber composites gradually increased. When the dosage of KH550 was 2 phr, the H pull-out force was the highest (109.0 ± 2.5) N. The new dipping system was able to achieve 106.7% of the RFL (102.2 ± 3.3) N/piece dipping system. The highest 180° peel force (23.5 ± 0.7) N/piece was achieved when the dosage of KH550 was 2 phr. The new dipping system was able to achieve115.8% of the RFL (20.3 ± 0.2) N/piece dipping system. This represents an increase of 21.1% and 43.3% compared to the system without KH550, indicating that the introduction of KH550 significantly improved the interfacial adhesion between the PET fiber and rubber. To confirm the general applicability of the KG-SML system, adhesion performance tests were carried out on PET/NR composites. The findings demonstrate that the KG-SML system notably enhances the adhesion performance of PET/NR composites, in accordance with the test trends described in Section 3.4. Relevant test details for PET/NR composites are available in the Supplementary Materials. This is primarily because the fiber surface, pre-treated with the glycerol ether first bath, reacted further to generate more hydroxyl groups. The silane coupling agent then underwent condensation reactions with these hydroxyl groups to form stable C-O-Si covalent bonds, while also introducing amino active groups. This enhanced both the chemical activity and hydrophilicity of the fiber surface, enabling strong bonding with rubber during the subsequent second bath dipping. When the KH550 content exceeded 2 phr, both the H pull-out force and peel strength showed a decreasing trend. This can be attributed to excess KH550 molecules tending to condense with each other via Si-OH groups formed during hydrolysis, forming oligomers. This leads to uneven silane layer thickness or even the formation of micron-sized agglomerates, which is detrimental to cord/rubber adhesion.

**Figure 7:**
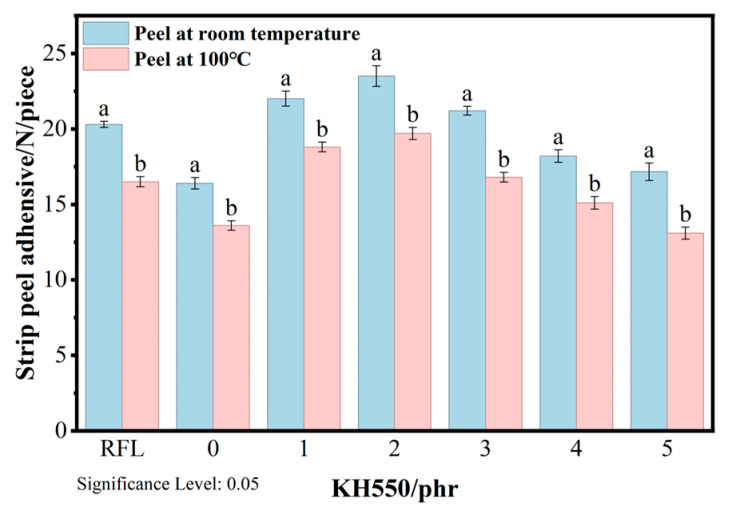
Strip peel adhesion at 100 °C (Lowercase letters a and b indicate a significant difference between the two sets of data obtained under different test conditions).

Given that FRRC are primarily used in high-temperature environments and require long service life, we will investigate the changes in adhesion properties under elevated temperatures and after aging. First, high-temperature peel tests were conducted to evaluate the adhesion between fibers and the rubber matrix under such conditions. Figure 7 shows the peel force of different specimens at various temperatures. The traditional RFL dipping system exhibited a 21.1% decrease in peel force at high temperatures, while the KG-SML system with 1 phr of KH550 showed the smallest reduction in peel force value, at 15.5%. This may be attributed to the curing agent promoting cross-linking reactions within the epoxy resin, resulting in a more stable network structure and improved interfacial adhesion between the fibers and their impregnation layers. However, excessive amounts of KH550 can lead to the over-amination of PET fibers, compromising their tensile strength and causing a significant decline in high-temperature peel force values.

**Figure 8:**
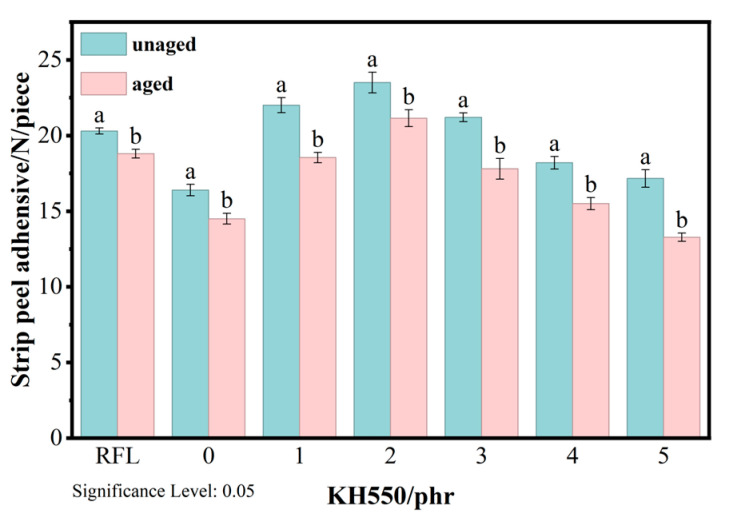
Aging stability of PET/SBR composites (Lowercase letters a and b indicate a significant difference between the two sets of data obtained under different test conditions).

Since the aging of FRRC materials can affect both interfacial adhesion and the intrinsic properties of the rubber itself, we conducted hot air aging tests on the specimens under the condition of 100 °C for 48 h. Figure 8 shows the peel force of the composite material decreased after aging, with the smallest reduction in peel force (11.3%) observed at a KH550 dosage of 2 phr. In comparison, the traditional RFL dipping system exhibited an 11.7% decrease in peel force after aging. Overall, the peel strength retention rate ranged between 78.4% and 88.7%, indicating that the samples maintained good adhesion performance even after thermal aging.

### 3.5. The Breaking Strength of Dip-Coated Fibers

**Figure 9:**
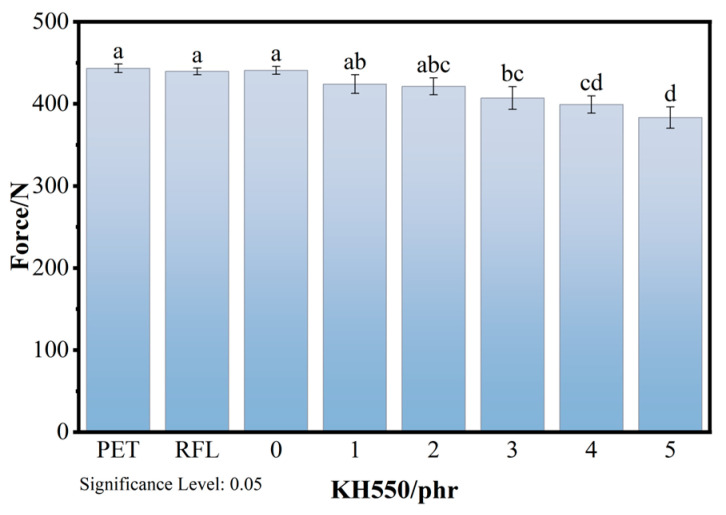
The breaking strength of PET fibers (Same lowercase letters indicate no significant difference between two groups of data, while different lowercase letters indicate a significant difference between them).

**Figure 10:**
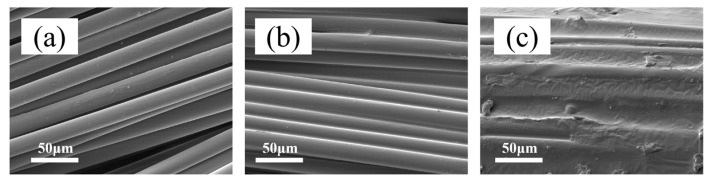
SEM images of fiber surface before and after impregnation: (**a**) unimpregnated PET fiber; (**b**) PET-KG fiber; (**c**) PET-KG-S fiber.

To investigate the effect of the silane coupling agent treatment on the mechanical properties of the polyester fibers, the tensile strength of the polyester cords before and after dipping was tested, with the results shown in Figure 9. As shown in Figure 9, after the introduction of the silane coupling agent, the tensile force of the fibers decreased to some extent with increasing KH550 content. Due to the reaction between the amino groups in KH550 and the ester groups in PET, PET undergoes ammonolysis. Additionally, a coating layer forms on the fiber surface after impregnation with the dipping solution, which can easily lead to stress concentration and tends to reduce the strength of PET. We can observe in Figure 10c that the PET surface was covered with a coating. However, the decrease in fiber tensile force was less than 5%, indicating that the influence of the dipping solution on the mechanical properties of the fiber is negligible. In other words, this dipping process does not adversely affect the industrial application of polyester fibers in rubber.

### 3.6. Surface Morphology of Fibers Before and After Dipping

The surface morphology of PET fibers before and after dipping was observed using SEM. Figure 10 shows SEM images of the untreated PET fiber, the fiber after the first bath treatment, and the fiber after the second bath treatment. From Figure 10a, it can be seen that the surface of the untreated PET fiber is smooth, resulting in only weak van der Waals forces between fiber bundles, presenting a loose and dispersed state. This structural feature makes the fibers prone to slippage during subsequent composite processing, hindering the formation of effective interfacial bonding. After the first dipping step, a thin epoxy impregnation layer appeared on the fibers (Figure 10b), and obvious physical connections began to form between the fiber bundles. Figure 10c shows that the fiber surface was covered by a thicker and denser impregnation layer. This would have mainly been due to the curing reaction of the epoxy resin on the fiber surface at a high temperature during the second treatment, forming a three-dimensional crosslinked network structure. Simultaneously, latex particles filled the gaps in the resin. This composite structure of the resin skeleton–latex reinforcement retained the high modulus characteristics of the epoxy resin while mitigating the modulus difference between the fiber and rubber through the elastic deformation capability of the latex particles, thereby significantly enhancing the interfacial adhesion performance.

### 3.7. Micro-Morphology of the Dip-Coated Fibers

**Figure 11:**
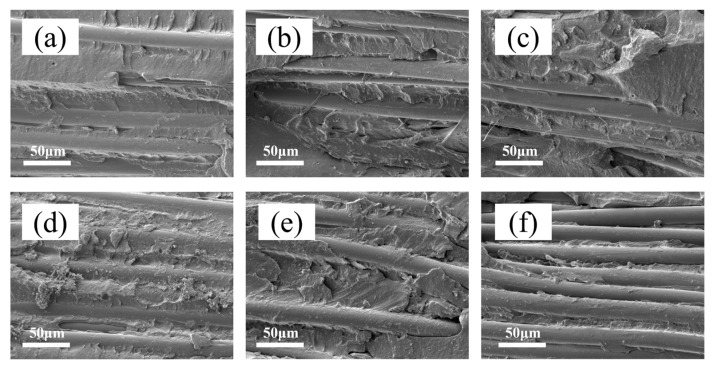
SEM images of the interface fracture surface of fiber/rubber composite materials after peel test: (**a**) KH550 = 0; (**b**) KH550 = 1; (**c**) KH550 = 2; (**d**) KH550 = 3; (**e**) KH550 = 4; (**f**) KH550 = 5.

The surface morphology of the fiber/rubber composites modified by the KG-SML dipping system after the 180° peel tests was observed using SEM. Figure 11 shows SEM images of the peeled surface morphology for composites with KH550 contents of 0, 1, 2, 3, 4 and 5 phr. It can be observed from Figure 11 that the peeled fiber surfaces all have a significant amount of rubber adhered, indicating that interfacial failure occurred within the rubber matrix itself rather than within the impregnation layer. This demonstrates that the KG-SML dipping system effectively enhanced the interfacial bonding, and the strength of the impregnation layer was sufficient to resist damage during the peeling process. Simultaneously, it can be observed that as the KH550 content increased, the amount of rubber covering the fiber surface after the peel test first increased and then decreased. When the KH550 content exceeded 2 phr, the amount of rubber adhered to the fiber surface after peeling decreased, which is consistent with the measured adhesion strength results.

### 3.8. Surface Elemental Analysis of Composites

**Figure 12:**
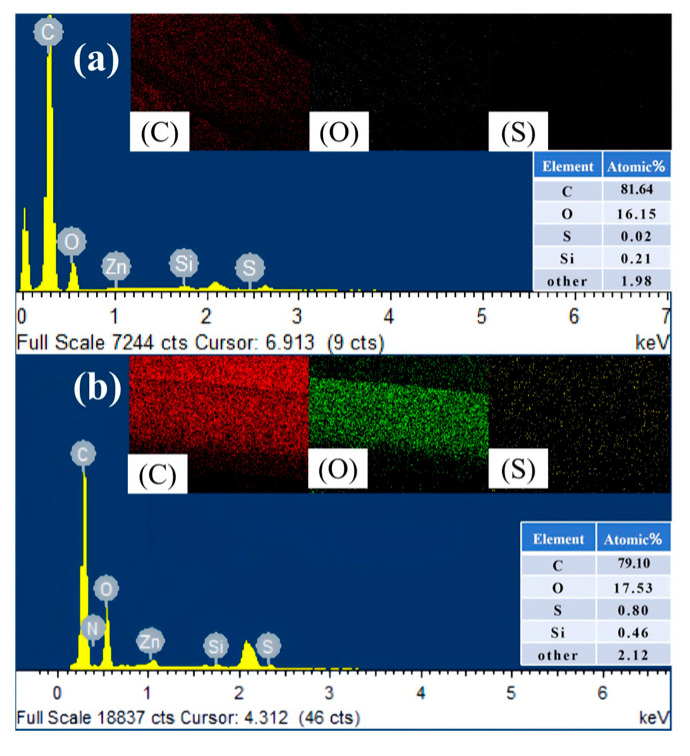
EDS analysis of the element content in the impregnated layer on the fiber surface: (**a**) before the vulcanization process; (**b**) after the vulcanization process.

To investigate the co-vulcanization mechanism between the impregnation layer and the rubber matrix, Energy Dispersive X-ray Spectroscopy (EDS) was performed on the fiber surface after the peel test of the composite rubber, as shown in Figure 12. The sulfur (S) element was barely present in the impregnation layer before co-vulcanization with the rubber matrix (Figure 12a). However, after vulcanization, the S element appeared on the surface of the impregnation layer, providing direct evidence that sulfur migrated from the rubber matrix into the impregnation layer (Figure 12b). During the vulcanization process, the VP latex and the rubber matrix underwent co-crosslinking reactions under the action of sulfur, forming crosslinks, which effectively enhanced the interfacial adhesion between the polyester fiber and rubber. Simultaneously, the silicon (Si) element was observed on the fiber surface, further verifying the successful grafting of KH550 onto the fiber surface.

## 4. Conclusions

To further enhance the interfacial adhesion between PET fibers and SBR, this study investigates adhesion improvement strategies based on an environmentally friendly dipping system. Two approaches were adopted: activating PET fibers with silane coupling agents and reinforcing them with strengthening agents. It was found that KH550 yielded the best modification effect, leading to the development of a novel KG-SML dipping system, in which KH550 was introduced in a one-bath process. When the KH550 dosage was 2 phr, both the peel force and H-pull force reached their maximum values, at (23.5 ± 0.7) N/piece and (109 ± 2.5) N, respectively. SEM observations revealed that the dipping treatment formed a dense and stable adhesive layer on the fiber surface, and peel failure of the composite occurred within the rubber matrix rather than the adhesive layer, indicating strong interfacial bonding between the fiber and rubber. EDS analysis indicated sulfur migration within the KG-SML adhesive layer, with sulfur enrichment at the fiber–rubber interface, promoting co-crosslinking between the latex components and the rubber matrix. This provides a microscopic explanation of the adhesion mechanism. From an industrial production perspective, the KG-SML system demonstrates distinct advantages over conventional RFL systems. It obviates the use of hazardous raw materials, which present notable risks to both human health and environmental safety. Furthermore, the system dispenses with the need for prolonged reaction durations, enabling a more simplified production process. This streamlined workflow not only cuts down on production time and costs substantially but also boosts overall operational efficiency in a tangible manner. The strength loss of the modified polyester fibers was controlled within 5%, demonstrating promising potential for industrial application.

## Figures and Tables

**Table 1:** Rubber compound composition.

Component	Content (Parts per Hundred of Rubber (phr))
SBR	100
Stearic acid	2
ZnO	5
Antiager 4010NA	1
Antiager RD	1.5
Carbon black N220	40
Sulfur	1
Accelerant CZ	1
Accelerant DTDM	1

## Data Availability

The original contributions presented in this study are included in the article. Further inquiries can be directed to the corresponding author.

## Supplementary Materials

The following supporting information can be downloaded at: https://www.mdpi.com/article/10.3390/polym18030338/s1, Figure S1: (a) H pull-out test and (b) 180° peel test of PET/ NR composites with different KH550 contents; Table S1: Rubber compound composition.

## Abbreviations

The following abbreviations are used in this manuscript:

FRRC	Fiber-reinforced rubber composites
PET	Polyester
RFL	Resorcinol–formaldehyde–latex
KH550	γ-Aminopropyltriethoxysilane
KG-SML	KH550-glycerol triglycidyl ether-sorbitol glycidyl ether-2-ethyl-4-methylimidazole-latex
phr	Parts per hundred of rubber
EPA	Environmental Protection Agency
IARC	International Agency for Research on Cancer
GTE	Glycerol triglycidyl ether
MZ	2-ethyl-4-methylimidazole
SGE	Sorbitol glycidyl ether
Na-MMT	Sodium montmorillonite
