# Supplementary material for: High Interfacial Adhesion of PET/Rubber Composites by a New Eco-Friendly Dipping System
Source: Polymers (Basel). 2026 Jan 27;18(3):338. doi: 10.3390/polym18030338 (PMC12899391; doi:10.3390/polym18030338)
Supplement: Supplementary file 1 [file polymers-18-00338-s001.zip › polymers-4110606-supplementary.pdf]

Supplementary Materials

1.Adhesion tests between PET and NR.

1.1. Materials

The composition of the NR compound employed in this study is detailed in Table S1.

Table S1. Rubber compound composition.

Component	Content (phr)
NR	100
Stearic acid	2
ZnO	4
Antiager 4020	1.5
Carbon black N330	40
Sulfur	2.5
Accelerant M	0.8
Aromatic oil	1.5

1.2. Preparation of PET fiber/rubber composites

The detailed procedures for sample fabrication are described in detail in Section 2 of the manuscript. The vulcanization temperature was set at 160℃. Each test uses five samples.

1.3. Influence of KH550 content on the static adhesion properties of PET/NR composites

Figure S1 reveals the influence of KH550 content on the adhesion properties of the KG-SML dipping system coated PET/NR composites. As shown, with increasing KH550 content, the H pull-out force and peel force of the KG-SML system modified fiber/rubber composites gradually increased, When the dosage of KH550 is 2phr, the H pull-out force and the 180° peel force was the highest. This result is in good agreement with the findings reported in the main text, thus confirming the general applicability of the KG-SML system.

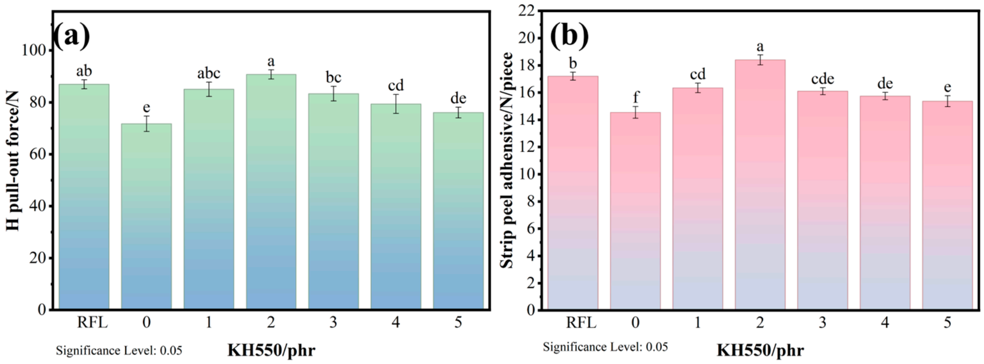

Figure S1. (a) H pull-out test and (b) 180 ° peel test of PET/ NR composites with different KH550 contents(Same lowercase letters indicate no significant difference between two groups of data, while different lowercase letters indicate a significant difference between them).
